# Unveiling the Interplay of EBV, HSV-1, and Inflammatory Biomarkers in Psychiatric Disorders

**DOI:** 10.3390/jcm14196730

**Published:** 2025-09-24

**Authors:** Özer Akgül, Ömer Faruk Demirel, İlker Tosun, Yasin Kavla, Mehmet Murat Kirpinar, Burcu Sapmaz, Gülçin Şenyiğit, Reyhan Çalişkan, Yaşar Ali Öner

**Affiliations:** 1 Department of Medical Microbiology, Faculty of Medicine, Istanbul Health and Technology University, 34275 Istanbul, Türkiye; 2 Department of Psychiatry, Cerrahpaşa Faculty of Medicine, Istanbul University—Cerrahpaşa, 34098 Istanbul, Türkiye; ofdmed@yahoo.com (Ö.F.D.); yasinkavla@gmail.com (Y.K.); muratkirpinar@gmail.com (M.M.K.); 3 Tesu Health Ltd., Cambridge CB1 3NH, UK; ilkertosun@hotmail.com; 4 Department of Medical Microbiology, Faculty of Medicine, Istanbul Aydın University, 34295 Istanbul, Türkiye; burcusapmaz@aydin.edu.tr (B.S.); yasaroner@aydin.edu.tr (Y.A.Ö.); 5 Vocational School of Health Services, Istanbul Aydın University, 34295 Istanbul, Türkiye; gulcinsenyigit@aydin.edu.tr; 6 Department of Medical Microbiology, Faculty of Medicine, Samsun University, 55030 Samsun, Türkiye; reyhan.caliskan@samsun.edu.tr

**Keywords:** psychiatric disorders, Epstein–Barr Virus (EBV), Herpes Simplex Virus type 1 (HSV-1), inflammatory biomarkers, immunopsychiatry

## Abstract

**Background/Objectives:** Schizophrenia (SCH), bipolar disorder (BPD), and major depressive disorder (MDD) are increasingly viewed as neuroimmune disorders shaped by viral exposure and inflammation. Disorder-specific immunovirological profiles, however, remain poorly defined. **Methods:** In this cross-sectional study, we assessed Epstein–Barr Virus (EBV) and Herpes Simplex Virus type 1 (HSV-1) seropositivity and measured serum CRP, IL-6, and IL-1β in 708 participants: 110 with SCH, 121 with BPD, 135 with MDD, and 342 healthy controls (HC). Statistical analyses included Shapiro–Wilk tests for normality; Kruskal–Wallis with Bonferroni-adjusted Dunn post hoc comparisons; and logistic regression adjusted for age, sex, and marital status. **Results:** EBV seropositivity was higher in SCH (90.9%) than in HC (78.9%) (OR = 3.46, 95% CI: 1.68–7.12; *p* = 0.001) but not in BPD or MDD. HSV-1 seropositivity was elevated in BPD (83.5%) versus HC (67.0%) (OR = 2.29, 95% CI: 1.34–3.92; *p* = 0.003), with no differences in SCH or MDD. Inflammatory biomarkers were significantly increased in SCH and MDD compared to HC (*p* < 0.001), while BPD showed no differences. **Conclusions:** The findings delineate distinct immunovirological patterns across major psychiatric disorders. Schizophrenia was characterized by EBV seropositivity accompanied by systemic inflammatory activation, bipolar disorder by HSV-1 seropositivity in the absence of inflammatory changes, and major depressive disorder by inflammatory dysregulation independent of viral exposure. These disorder-specific profiles highlight heterogeneity in neuroimmune pathways and underscore the potential relevance of biomarker-based stratification for generating hypotheses regarding targeted antiviral or anti-inflammatory interventions in psychiatric populations.

## 1. Introduction

Psychiatric disorders, encompassing schizophrenia (SCH), bipolar disorder (BPD), and major depressive disorder (MDD), represent significant contributors to global disability, substantially affecting emotional, cognitive, and social dimensions of functioning [1,2]. These conditions are characterized by a multifaceted etiology, influenced by a combination of genetic predispositions, environmental factors, and biological mechanisms [3,4]. Recent decades have seen an amplified focus on the role of immune system irregularities, notably neuroinflammation and viral infections, in the underlying mechanisms of psychiatric disorders [5,6,7].

While *Toxoplasma gondii* is frequently cited as a potential etiological factor in SCH [8], BPD, and MDD [9], it is important to note that viral infections have also been theorized to contribute to psychiatric disorders through immune system activation and consequent neuroinflammation. In addition, EBV and HSV-1 were prioritized because they are neurotropic, highly prevalent, and consistently implicated in neuroimmune alterations relevant to major psychiatric disorders. By contrast, associations for other pathogens such as CMV or *Toxoplasma gondii* are more heterogeneous across studies [10,11]. Viral infections may indeed contribute to psychiatric disorders through the activation of the immune system, subsequently leading to inflammation within the brain. Notably, the Epstein–Barr Virus (EBV), a member of the Herpesviridae family, has been specifically implicated in the development of schizophrenia and related psychiatric conditions [12]. Evidence suggests that EBV can induce persistent neuroinflammation, potentially contributing to cognitive dysfunction and the manifestation of psychiatric symptoms [13]. Research indicates that individuals with schizophrenia exhibit elevated levels of EBV-specific IgG antibodies compared to controls, suggesting an altered immune response [14]. The association between EBV and schizophrenia may be related to the virus’s ability to induce persistent neuroinflammation, which is thought to contribute to cognitive dysfunction and symptomatology in affected individuals [15].

Similarly, Herpes Simplex Virus (HSV), particularly HSV-1, has been associated with cognitive impairments in SCH. Studies show that HSV-1 seropositivity correlates with negative symptoms and cognitive deficits, suggesting that the virus may contribute to the neurobiological abnormalities observed in this disorder [16]. The neuroinflammatory effects of HSV-1 infection further highlight the role of viral agents in the exacerbation of psychiatric symptoms [17,18]. Although the precise mechanisms through which these viral infections contribute to psychiatric pathologies remain under investigation, current hypotheses posit direct neural interference or immune-mediated pathways involving elevated cytokine levels and altered gene expression [19]. Nissen and colleagues posited that HSV-1 infection is significantly associated with suicidal behavior and could potentially serve as a causal factor for various psychiatric disorders, given that HSV-1 infection frequently precedes the initial psychiatric diagnosis [20].

Beyond the scope of viral etiologies, chronic inflammation may offer additional insights into the complex pathophysiology of psychiatric disorders [21]. Consequently, consistently elevated levels of inflammatory markers, including C-Reactive Protein (CRP), Interleukin-6 (IL-6), and Interleukin-1β (IL-1β), have been noted in individuals diagnosed with SCH, BPD, and MDD, signifying the presence of systemic inflammation [22]. CRP, a commonly measured inflammatory marker, shows notable elevation in individuals with major depressive disorder, particularly those with severe manifestations of the condition [23]. This elevation in CRP levels is correlated with both the intensity of symptoms and unfavorable prognoses, pointing to its utility as a potential biomarker for gauging disease activity [23]. Moreover, IL-6 and IL-1β have been implicated in the inflammatory mechanisms that underlie psychiatric disorders. Research has indicated that increased IL-6 levels are related to a heightened inflammatory response within the brain, fostering microglial activation and the worsening of psychiatric symptoms [22]. Similarly, elevated IL-1β levels are frequently seen in both acute and chronic psychiatric conditions, further substantiating the significance of inflammation in the pathophysiology of these diseases [22]. These biomarkers were chosen because they are consistently replicated in meta-analyses of psychiatric disorders, showing robust associations with schizophrenia and depression. While other markers such as TNF-α and microglial activation markers have been studied, findings remain inconsistent and phase-specific. Therefore, CRP, IL-6, and IL-1β were prioritized as reliable indicators of systemic inflammation.

This study endeavors to investigate EBV, HSV-1, CRP, IL-6, and IL-1β in the blood of patients diagnosed with SCH, BPD, and MDD, relative to healthy controls (HC). The central goal is to evaluate the potential involvement of these biomarkers in the pathophysiology of these psychiatric conditions, with specific attention to the interplay between viral infections, inflammatory responses, and the intensity of the disease. Through the analysis of these markers, this research aims to clarify the significance of immune system dysregulation in the etiopathogenesis and advancement of psychiatric illnesses, as well as to pinpoint possible biomarkers beneficial for diagnostic and therapeutic strategies.

## 2. Materials and Methods

### 2.1. Study Design and Participants

**Figure 1:**
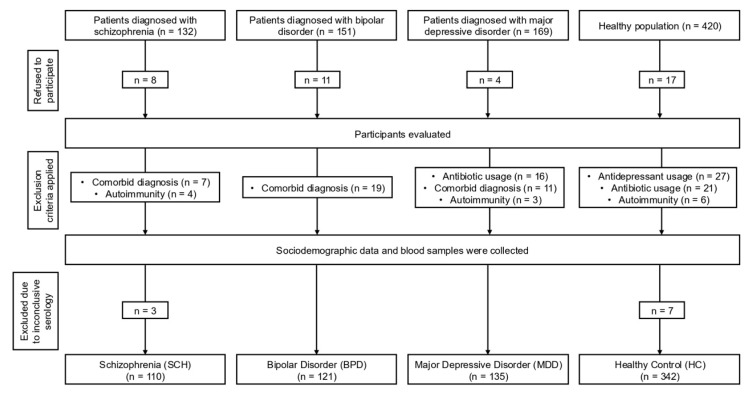
Flowchart depicting the stages of this cross-sectional study.

This cross-sectional study aimed to evaluate the seropositivity of EBV and HSV-1, alongside the levels of CRP, IL-6, and IL-1β in individuals diagnosed with SCH, BPD, and MDD, in comparison to HC. Prior to commencing the research, the Istanbul Aydin University Clinical Research Ethics Committee approved this study (#480.2/217). The study sample consisted of 708 participants, categorized into the following groups: 110 individuals diagnosed with SCH, 121 with BPD, 135 with MDD, and 342 HC. Participants aged 18–62 years were included in the study. To ensure the homogeneity of the sample, exclusion criteria were implemented, specifically excluding individuals with comorbid psychiatric diagnoses based on the Structured Clinical Interview for Diagnostic and Statistical Manual of Mental Disorders, Fifth Edition (DSM-5), active infections, autoimmune or inflammatory conditions, and current or previous use of immunosuppressant therapies. SCH, BPD, and MDD groups were recruited from the inpatient and outpatient facilities of the Department of Psychiatry at Istanbul University—Cerrahpaşa, based on the diagnostic criteria specified in the DSM-5. For clarity regarding the research approach employed, a flow diagram delineating the sequential stages of the study is provided (Figure 1).

### 2.2. Sample Collection and Serological Analysis

Blood samples were obtained from all participants to evaluate EBV IgG and HSV-1 IgG seropositivity and to quantify CRP, IL-6, and IL-1β levels. Subsequently, serum samples were isolated and stored at −80 °C pending serological assays. The EBV IgG analysis was conducted using the Epstein–Barr Virus EBNA-1 IgG ELISA Kit (Abnova, Taipei City, Taiwan), for which the manufacturer reported a sensitivity of 100% and a specificity of 96.4%. The HSV-1 IgG analysis was conducted utilizing the HSV-1 IgG ELISA Kit (Atlas Medical, Berlin, Germany), which the manufacturer reported exhibits a sensitivity of 98.10% and a specificity of 99.48%. Final absorbance values were read at 450 nm, and the concentration of samples was evaluated. According to the manufacturers’ instructions, EBV IgG and HSV-1 IgG values below 0.90 were considered seronegative, while values above 1.10 were considered seropositive.

For inflammatory biomarkers analysis, CRP (The Invitrogen™Human C-Reactive Protein ELISA kit, #KHA0031, Thermo Fisher Scientific, Waltham, MA, USA), IL-6 (The Invitrogen™ Human IL-6 ELISA kit, #EH2IL6, Thermo Fisher Scientific, Waltham, MA, USA) and IL-1β (The Invitrogen™ Human IL-1β ELISA kit, #BMS224-2, Thermo Fisher Scientific, Waltham, MA, USA) levels were evaluated, and results are presented as pg/mL, following the manufacturer’s instructions.

### 2.3. Statistical Analysis

All statistical analyses were performed using IBM SPSS Statistics version 22.0 (IBM Corp., Armonk, NY, USA). The normality of CRP, IL-6, and IL-1β distributions was evaluated with the Shapiro–Wilk test. As all markers deviated from normality, non-parametric Kruskal–Wallis tests were applied for group comparisons. When appropriate, pairwise post hoc analyses were conducted using Dunn’s test with Bonferroni-adjusted significance threshold for six pairwise comparisons (α = 0.0083). Continuous variables are summarized as median with interquartile range (IQR) for inflammatory biomarkers and as mean ± SD for age; categorical variables (e.g., viral seropositivity) were compared using Pearson’s chi-square test. To examine independent associations between EBV or HSV-1 seropositivity and diagnostic groups, binary logistic regression models were fitted with healthy controls (HC) as the reference category, adjusting for age, sex, and marital status. Adjusted odds ratios (ORs) with 95% confidence intervals (CIs) are reported. A two-tailed ***p*** < 0.05 was considered statistically significant.

## 3. Results

The demographic composition of the study population across all groups is detailed in Table 1. Comparative analyses revealed a statistically significant difference in mean age between SCH and HC (*p* = 0.013), whereas age distributions in the BPD (*p* = 0.148) and MDD (*p* = 0.084) groups did not significantly deviate from that of controls. Sex distribution was comparable across most groups, with no significant differences observed between SCH (*p* = 0.917) or BPD (*p* = 0.497) and the HC. However, the MDD group exhibited a modest yet statistically meaningful shift in gender proportions (*p* = 0.047). Marital status distributions diverged significantly in the SCH (*p* < 0.001), BPD (*p* = 0.022), and MDD (*p* = 0.002) groups relative to HC.

**Figure 2:**
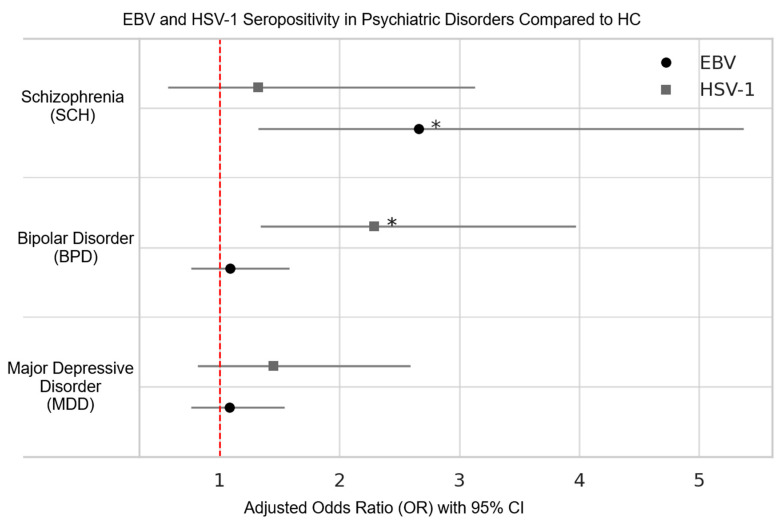
The adjusted OR and 95% CI for seropositivity of EBV and HSV-1 in psychiatric disorders compared to healthy controls. Statistical significance is denoted by an asterisk.

Serological profiling revealed differences for Epstein–Barr Virus (EBV) and Herpes Simplex Virus type 1 (HSV-1) across psychiatric groups (Table 2). Multivariable logistic regression analyses were performed to examine disorder-specific associations with EBV and HSV-1 seropositivity, using HC as the reference group and adjusting for age, sex, and marital status. Patients with schizophrenia had significantly increased odds of EBV IgG seropositivity (OR = 3.46, 95% CI: 1.68–7.12, *p* = 0.001), whereas no significant differences were observed for BPD (*p* = 0.632) or MDD (*p* = 0.662) compared with controls. In contrast, HSV-1 IgG seropositivity was significantly elevated in BPD (OR = 2.29, 95% CI: 1.34–3.92, *p* = 0.003), while neither SCH (*p* = 0.519) nor MDD (*p* = 0.207) differed from HC. Age emerged as a significant covariate in both viral models, whereas sex and marital status did not exert significant effects. Figure 2 depicts the adjusted ORs and 95% confidence intervals for EBV and HSV-1 for all groups.

**Figure 3:**
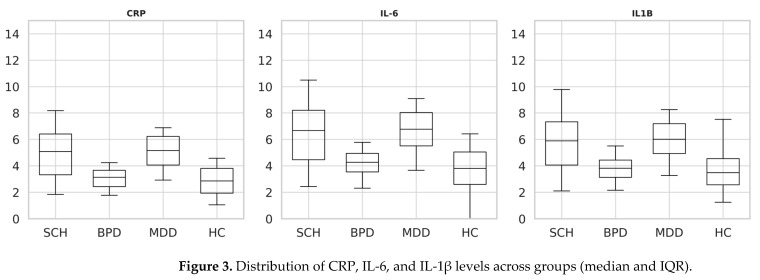
Distribution of CRP, IL-6, and IL-1β levels across groups (median and IQR).

Distributional assumptions were first evaluated using Shapiro–Wilk tests, which confirmed significant deviations from normality for all three markers (CRP, IL-6, IL-1β) across groups. Consequently, non-parametric analyses were applied. Kruskal–Wallis omnibus tests indicated highly significant overall group effects for CRP (H = 262.5, df = 3, *p* < 1 × 10^−55^), IL-6 (H = 259.5, df = 3, *p* < 1 × 10^−55^), and IL-1β (H = 262.1, df = 3, *p* < 1 × 10^−55^). Subsequent Bonferroni-adjusted Dunn post hoc tests (α = 0.0083) revealed that both SCH and MDD exhibited significantly elevated concentrations of CRP, IL-6, and IL-1β relative to HC (all adj. *p* < 0.001). In contrast, BPD did not differ from HC on any biomarker (all adj. *p* = ns). SCH and MDD did not differ from one another (all adj. *p* = ns), whereas BPD demonstrated significantly lower levels of all inflammatory markers compared with both SCH and MDD (all adj. *p* < 0.001). No significant differences in IL-1β levels were observed between BPD and HC following Bonferroni-adjusted post hoc comparisons. Median values with interquartile ranges (IQR) and significant contrasts are reported in Table 3. Figure 3 illustrates boxplot distributions (median and IQR) for each marker across groups, consistent with the non-parametric analytic framework.

## 4. Discussion

The current investigation explored viral exposure and inflammatory responses across schizophrenia, bipolar disorder, and major depressive disorder compared to HC. The study revealed distinct immunovirological and inflammatory signatures, further supported by comparisons with current meta-analytical evidence. In addition, schizophrenia and depression are multifactorial disorders shaped by genetic susceptibility, environmental exposures, stress biology, and timing of infection; these factors were not fully captured here. Other neurotropic pathogens (e.g., CMV) and immune markers were not assessed and may contribute to observed patterns. For bipolar disorder, HSV-1 has been linked to cognitive and affective changes through neurotropic mechanisms even in the absence of systemic inflammation [23]. For major depressive disorder, evidence remains mixed, with reports of bidirectional associations in viral reactivation contexts as well as null findings [10,24]. Antidepressant use, which we did not measure, may also confound associations. Overall, schizophrenia and depression are multifactorial conditions shaped by genetic, environmental, and stress-related factors, and other pathogens such as CMV may contribute [11].

A salient observation in our cohort was the higher Epstein–Barr Virus (EBV) seropositivity among patients with schizophrenia; in adjusted models, schizophrenia showed approximately 2.7-fold higher odds relative to healthy controls (OR = 2.66; *p* = 0.006), whereas no elevation was observed in bipolar disorder or major depressive disorder. In line with this nuanced pattern, Dickerson et al. [12] reported an odds ratio of 8.86 (95% CI: 2.59–30.37; *p* < 0.001) only among individuals who combined high EBV-VCA titers with elevated polygenic risk scores for schizophrenia—indicating an interaction between viral immune response and genetic susceptibility rather than a generalized effect of EBV seropositivity. While prior work has posited that heightened VCA—but not EBNA-1—responses may reflect latent or reactivated EBV with downstream neuroimmune consequences [12], our IgG-based serology cannot distinguish prior from reactivated infection. Finally, although one cross-sectional/Mendelian-randomization study reported a small effect estimate linking EBV exposure with major depressive disorder [25], our data showed no herpesvirus associations in MDD; together with our cross-sectional design, these findings should be interpreted as associational and hypothesis-generating, not causal.

Recent mechanistic insights reinforce the plausibility of EBV as a contributor to neuropsychiatric pathology. Wang et al. (2022) demonstrated that EBV infection of human glial cells induces aberrant inflammatory cascades, particularly up-regulation of IL-6 and TNF-α, which have been implicated in neurotoxicity and cognitive decline in schizophrenia [26]. In parallel, Runge et al. (2022) detected elevated EBV antibody indices in cerebrospinal fluid of schizophrenia spectrum patients, suggesting active or reactivated viral presence within the CNS [15]. Moreover, the review by Kotsiri et al. (2023) emphasized the cumulative neurodevelopmental risks posed by early-life EBV exposure, highlighting its potential role in microglial sensitization and synaptic pruning deficits implicated in psychosis [19]. Additional support arises from a recent PET imaging meta-analysis, which confirmed microglial overactivation in schizophrenia, predominantly in frontotemporal regions—consistent with EBV-related immune perturbation pathways [21]. Collectively, this evidence underlines the need for further investigation of EBV-targeted diagnostics and personalized immunomodulatory approaches in psychiatric populations.

HSV-1 seropositivity was elevated solely in BPD patients, without concurrent inflammatory biomarker increases. The prior literature has primarily linked HSV-1 with cognitive impairment rather than overall seroprevalence in schizophrenia. Indeed, a meta-analysis by Snijders et al. (2019) reported moderate effect sizes (Cohen’s d ≈ −0.23 to −0.49) for memory and attention deficits associated with HSV-1 in schizophrenia and bipolar groups [10]. In bipolar disorder, Dickerson et al. (2004) first described HSV-1 seropositivity as an independent predictor of verbal memory impairment (F = 12.07; *p* < 0.001), and subsequent randomized trials demonstrated cognitive improvement following valacyclovir administration—53% of seropositive BPD patients showed ≥10-point RBANS gain after four months of antiviral therapy, compared to 14% in placebo [23].

Although a meta-analysis comprising 2364 BPD cases did not find elevated HSV-1 seroprevalence (OR = 0.84; 95% CI: 0.57–1.23) [10], the divergent results may reflect factors such as mood state, genetic predisposition, or regional seroepidemiology. Moreover, a 2025 cohort study revealed a bidirectional relationship between herpesvirus reactivation and major depressive disorder, suggesting that HSV-1 reactivation may also exacerbate affective symptoms through immune-mediated mechanisms [27]. Mechanistic studies provide further support. A recent mBio animal study demonstrated that intranasal HSV-1 inoculation induces long-term neurobehavioral deficits, including anxiety and memory impairment, via heparanase-dependent neuroinflammation [28]. A 2023 review emphasized that cognitive impairment in BPD is predominantly observed in processing speed, attention, and executive function—even during euthymic states—suggesting enduring neurocognitive dysfunction independent of mood episodes. These domains notably overlap with those affected in HSV-1 seropositive individuals, where viral latency and reactivation have been implicated in persistent deficits in working memory and cognitive flexibility [29]. Taken together, these findings suggest that while general HSV-1 seropositivity may not differ between BPD and controls, viral reactivation and underlying neurotropic injury—potentially mediated via inflammation and neuronal enzyme pathways—appear to contribute to cognitive dysfunction in susceptible individuals.

Our findings demonstrated significant elevations in CRP, IL-6, and IL-1β levels among individuals with schizophrenia (SCH) and major depressive disorder (MDD), whereas no significant differences were observed in bipolar disorder (BPD). In schizophrenia, median CRP, IL-6, and IL-1β concentrations were significantly higher than in healthy controls (all adj *p* < 0.001, Kruskal–Wallis with Bonferroni correction), in agreement with a meta-analysis of 58 studies reporting a pooled standardized mean difference (SMD) of 0.53 (95% CI: 0.30–0.76) for CRP levels compared to controls [30]. IL-6 elevations (median 6.67 pg/mL vs. 3.81 pg/mL in controls) paralleled this trend (adj *p* < 0.001), consistent with a meta-analytic SMD of 0.44 (95% CI: 0.34–0.55) across clinical phases [30]. IL-1β was likewise significantly increased in SCH (median 5.89 pg/mL vs. 3.48 pg/mL in controls; adj *p* < 0.001), corroborating cumulative evidence of its role in neuroinflammatory pathogenesis [28]. These results strengthen the view that schizophrenia is associated with chronic low-grade systemic inflammation that may contribute to neurocognitive deficits and illness progression.

In MDD, all three inflammatory markers were significantly elevated compared with controls (CRP median 6.81 mg/L, IL-6 8.69 pg/mL, IL-1β 7.79 pg/mL; all adj *p* < 0.001). These findings support the concept of an inflammatory subtype of major depressive disorder. Our results align with meta-analyses reporting SMDs of 0.71 for CRP, 0.61 for IL-6, and 0.35 for IL-1β [25,31]. They are further reinforced by Mendelian randomization analyses suggesting causal relationships between systemic inflammation and major depressive phenotypes [25,32]. The reproducibility of these inflammatory elevations across cohorts and methods reinforces the concept of “inflammatory depressive disorder,” a promising target for adjunctive anti-inflammatory therapies.

In contrast, BPD patients did not differ significantly from controls for any biomarker (all adj *p* = NS). While some studies have reported episodic cytokine dysregulation (e.g., SMDs of 0.70 for CRP and 0.81 for IL-6), these elevations are typically phase-specific—pronounced during acute mania or depressive episodes and attenuated during euthymia [33]. The absence of significant elevations in our predominantly euthymic sample (CRP, IL-6, IL-1β all non-significant vs. controls) underscores the transient and state-dependent nature of inflammatory changes in BPD. These disorder-specific immunoinflammatory trajectories suggest that SCH and MDD are characterized by sustained immune dysregulation, whereas BPD reflects episodic fluctuations, highlighting the importance of temporal precision in biomarker sampling.

This study has several limitations. First, its cross-sectional design precludes causal inference regarding the temporal relationship between viral exposure, inflammatory dysregulation, and psychiatric onset. Second, the serological assessment relied exclusively on IgG positivity, which cannot differentiate past, latent, and reactivated infections. More precise approaches such as PCR-based viral detection, CSF antibody indices, or viral DNA quantification should be incorporated in future studies. Third, although we adjusted for age, sex, and marital status, other potential confounders—including medication use, smoking, alcohol consumption, BMI, and metabolic conditions—were not assessed. Fourth, resource constraints limited the breadth of biomarkers and advanced virological assays, restricting our immune panel to CRP, IL-6, and IL-1β. Finally, while the sample size was sufficient to detect moderate effects, smaller associations may have gone undetected.

## 5. Conclusions

In conclusion, this work delineates disorder-specific immunovirological patterns across major psychiatric conditions. Schizophrenia was marked by the convergence of heightened EBV seropositivity and systemic inflammatory activation; bipolar disorder by an increased prevalence of HSV-1 seropositivity in the absence of inflammatory elevation; and major depressive disorder by inflammatory dysregulation independent of herpesvirus exposure. These patterns underscore correlational rather than causal associations and should be interpreted within the constraints of a cross-sectional design. Nevertheless, they suggest that stratification by immune and viral biomarkers may provide a valuable framework for generating mechanistic hypotheses and guiding the design of future longitudinal and interventional studies targeting neuroimmune pathways in psychiatry.

## Figures and Tables

**Table 1:** Sociodemographic characteristics of the study participants.

		Schizophrenia (SCH)	*p*-Value vs. HC	Bipolar Disorder (BPD)	*p*-Value vs. HC	Major Depressive Disorder (MDD)	*p*-Value vs. HC	Healthy Control (HC)
Age (Mean ± SD)		36.88 ± 12.74	**0.013**	42.24 ± 12.10	0.148	42.48 ± 11.80	0.084	40.37 ± 12.51
Gender n (%)	Female	46 (42%)	0.917	57 (47%)	0.497	44 (33%)	**0.047**	147 (43%)
	Male	64 (58%)		64 (53%)		91 (67%)		195 (57%)
Marital Status n (%)	Single	71 (65%)	**<0.001**	64 (53%)	**0.022**	76 (56%)	**0.002**	138 (40%)
	Married	39 (35%)		57 (47%)		59 (44%)		204 (60%)

**Table 2:** Adjusted EBV and HSV-1 Seropositivity in Psychiatric Disorders vs. HC.

	EBV			HSV-1		
	Seropositivity n (%)	OR (95% CI)	*p*-Value vs. HC	Seropositivity n (%)	OR (95% CI)	*p*-Value vs. HC
**Schizophrenia (SCH)**	100/110 (90.9%)	3.46 (1.68–7.12)	**0.001**	76/110 (69.1%)	0.88 (0.57–1.13)	0.519
**Bipolar Disorder (BPD)**	98/121 (81.0%)	1.12 (0.76–1.65)	0.632	101/121 (83.5%)	2.29 (1.34–3.92)	**0.003**
**Major Depressive Disorder (MDD)**	109/135 (80.7%)	0.98 (0.76–1.54)	0.662	91/135 (67.4%)	1.12 (0.82–2.58)	0.207
**Healthy Control (HC)**	270/342 (78.9%)	Reference	NA	229/342 (67.0%)	Reference	NA

Odds ratios (OR) and 95% confidence intervals (CI) derived from binary logistic regression adjusted for age, sex, and marital status. Healthy controls (HC) served as the reference category. *p*-values are unadjusted.

**Table 3:** Serum inflammatory biomarkers across all groups with Kruskal–Wallis and Bonferroni-adjusted comparisons.

	Median (IQR)			Significant Contrasts (Bonferroni)
	CRP	IL-6	IL-1 β	
**Schizophrenia (SCH)**	5.08 (3.32–6.41)	6.67 (4.46–8.20)	5.89 (4.06–7.33)	**↑ vs. HC; ↓ vs. BPD; ≈MDD**
**Bipolar Disorder (BPD)**	3.14 (2.43–3.66)	4.26 (3.54–4.94)	3.82 (3.13–4.44)	**≈HC; ↓ vs. SCH & MDD**
**Major Depressive Disorder (MDD)**	5.16 (4.06–6.22)	6.77 (5.51–8.03)	6.01 (4.93–7.18)	**↑ vs. HC; ≈SCH**
**Healthy Control (HC)**	2.85 (1.93–3.81)	3.81 (2.60–5.05)	3.48 (2.57–4.55)	Reference
**Kruskal–Wallis** **H, (*p*)**	262.5 (***p* < 0.001**)	259.5 (***p* < 0.001**)	262.1 (***p* < 0.001**)	NA

Values are presented as median (interquartile range, IQR). Units: CRP (mg/L), IL-6 (pg/mL), IL-1β (pg/mL). Kruskal–Wallis tests indicated significant group differences for all markers. Pairwise comparisons were performed with Dunn’s test and Bonferroni correction (α = 0.0083). ↑ is indicated increase; ↓ is indicated decrease; ≈ is indicated similar.

## Data Availability

The datasets used in this study are available from the corresponding author upon reasonable request.
